# Home-based self-management for sedentary individuals with mild walking disability after stroke: protocol for a randomised pilot study

**DOI:** 10.1186/s12883-023-03461-7

**Published:** 2023-11-20

**Authors:** Maria Tereza Mota Alvarenga, Louise Ada, Elisabeth Preston, Lívia Cristina Guimarães Caetano, Luci Fuscaldi Teixeira-Salmela, Aline A Scianni

**Affiliations:** 1 Department of Physiotherapy, Federal University of Minas Gerais, Av. Presidente Antônio Carlos 6627- Pampulha, Belo Horizonte, 31270-901 Brazil; 2 Faculty of Medicine and Health, The University of Sydney, Sydney, Australia; 3 School of Rehabilitation and Exercise Science, University of Canberra, Canberra, Australia

**Keywords:** Stroke, Physical activity, Self-management, Behaviour change techniques, Home-based intervention, Pilot clinical trial

## Abstract

**Background:**

A Phase I study showed that it is feasible to implement a home-based self-management program aimed at increasing physical activity in individuals after stroke with mild walking disability in Brazil. The next step is to test this program against a control group in order to provide a power analysis for a fully-powered Phase III clinical trial.

**Methods:**

A Phase II pilot randomised clinical trial with concealed allocation, blinded measurement, and intention-to-treat analyses will be carried out. The inclusion criteria will be individuals diagnosed with stroke, in the acute or subacute phase, with mild walking disability, sedentary, and no significant language impairment. The participants will be randomly allocated to the experimental or control group. The experimental group will receive six sessions of a home-based self-management program based on behaviour change techniques through the Social-Cognitive Theory and Control Theory over 11 weeks. The control group will receive one session of education about stroke (regarding the importance of practising physical activity after a stroke) and usual care. A total of 24 participants will be recruited. The primary outcome will be physical activity, measured through steps taken per day by an activity monitor (Actigraph wGT3X-BT, Pensacola, FL, USA). The mean of daily steps will be analysed to compare groups after intervention. Secondary outcomes will be cardiovascular risk (body mass index, waist circumference, and blood pressure), depressive symptoms (Geriatric Depression Scale), walking ability (6-Minute Walk Test and 10-Meter Walk Test), exercise self-efficacy (Self-Efficacy for Exercise scale), social participation (Stroke Impact Scale) and quality of life (EuroQual-5D). Two-way analyses of variance will be implemented for all parametric outcomes, and the Kruskal–Wallis test for non-parametric outcomes will be used to determine the statistical significance of the between-group differences and reported as mean differences between groups (95% CI). All analyses will be conducted intention-to-treat. All outcomes will be measured at baseline (Week 0), post-intervention (Week 12), and follow-up (Week 24). This pilot clinical trial was registered online at Clinical Trials under number NCT05461976 on 4^th^ April 2022.

**Discussion:**

If beneficial, this Phase II pilot randomised trial will provide data to plan a fully powered future Phase III clinical trial aimed at verifying the efficacy of this program to promote physical activity after stroke.

**Trial registration:**

Clinical Trials NCT05461976 on 4^th^ April 2022.

**Supplementary Information:**

The online version contains supplementary material available at 10.1186/s12883-023-03461-7.

## Background

Keeping stroke survivors physically active is a challenge [1, 2]. Promoting physical activity after a brain lesion is essential to prevent recurrent events and associated risk factors [2]. However, stroke survivors face barriers in maintaining physical activity [1, 3]. *Behaviour change techniques* have been used after stroke to overcome these barriers [4]. Recently, a stroke guideline recommended that, in individuals with mild disability, implementing *behaviour change techniques* could have a positive impact on the level of physical activity [2].

One way to promote behaviour change after stroke is through self-management programs [4, 5]. These programs aim to help participants manage their condition by providing information and encouragement, which may be a good way of keeping stroke survivors physically active [5, 6]. There are cautious findings about the effectiveness of this strategy, suggesting the need for further studies in the area [4, 7, 8]. One review examined the evidence supporting self-management interventions for stroke survivors and reported some effectiveness in promoting physical activity [7]. Another review showed self-management programs based on *behaviour change techniques* were the most promising interventions to promote long-term free-living physical activity [4]. Finally, an integrative review describing the evidence around self-management interventions to improve mobility after stroke reported a deficient grade of evidence for the change in walking speed, endurance, balance, and physical activity (steps/day) [8].

Recently, we demonstrated the feasibility of implementing a home-based self-management exercise program to promote physical activity among stroke survivors with mild walking disability in Brazil [1]. Therefore, a Phase II trial is required to test this program against a control group to provide a power analysis for a fully-powered Phase III clinical trial. However, the feasibility analysis raised several essential points. First, the results revealed that sedentary participants, i.e., fewer than 5,000 steps per day, derived the most benefit from the program. Second, 59% of eligible individuals did not receive medical clearance to participate. Third, the primary barriers to physical activity were lack of support, knowledge, and accessibility. Fourth, only 28% of participants consistently filled in the diaries with information regarding their adherence to the program. Lastly, participants expressed that the measurement sessions were too long. A Phase II pilot randomised trial will address these issues and, in addition, we will answer the following questions:

(i) In sedentary individuals with a mild walking disability after stroke, does a home-based self-management exercise program promote physical activity (i.e., number of steps taken per day) more than usual care at 3 and 6 months?
(ii) Does any improvement in physical activity carry over to improvements in cardiovascular risk, walking ability, depressive symptoms, exercise self-efficacy, social participation, and quality of life?

## Methods

### Design

A prospective, pragmatic, two-arm, parallel-group, Phase II pilot randomised clinical trial with concealed allocation, allocation ratio (1:1), blinded measurement, and intention-to-treat analyses will be carried out. The study was approved by the Federal University of Minas Gerais Ethics Board (65672517.6.0000.5149) Hospital Ethics Board (17/2017) and registered on clinicaltrials.gov on 4^th^ April 2022 (NCT05461976). Any modifications that may occur during this trial will be reported in clinicaltrials.gov.

### Recruitment

Individuals who have suffered a mild stroke after being discharged from a public hospital in Belo Horizonte City, Brazil and fulfilling inclusion criteria will be invited to participate by a research team member. The hospital is a stroke referral and accepts all kinds of patients (e.g. all ages, types of stroke, and severity).

### Inclusion criteria

The inclusion criteria will be the same as those for the feasibility study. The only exception now is that we will only include sedentary participants, as the feasibility study showed they benefited most from the program. Therefore, the inclusion criteria are: (1) time since stroke less than six months (2) ≥ 18 years of age; (3) able to walk ten meters independently at a speed ≥ 0.8 m/s without any walking devices; [9] (4) no cognitive impairments (determined by the cut-off scores on the Brazilian version of the Mini-Mental State Examination); [10] (5) sedentary (mean of steps counts < 5,000 steps/day [11], over four days, determined by a triaxial accelerometer—Actigraph wGT3X-BT, Pensacola, FL, USA). The exclusion criteria are (1) other neurological diseases; (2) comprehensive aphasia (evaluated by simple motor command); [12] (3) any other conditions that would prevent participation (e.g., recent post-surgery or surgery scheduled, travel or further compromise that prevents the participant from staying in Belo Horizonte during the intervention period).

### Procedures

Before starting the study, a research team member will provide complete study information for eligible participants. Those who fulfil the inclusion criteria, agree to participate and have received medical clearance will be invited to provide written consent. In order to overcome the lack of medical clearance found in the feasibility study, clinicians (not members of the research team) will be informed about the study’s objectives and the importance of physical activity for secondary prevention and encouraged to refer stroke survivors to the trial. During the study, who will be responsible for the participants will be the research team. After screening for eligibility and providing written consent, participants will be randomly allocated to either the experimental (home-based self-management program and usual care) or the control group (education and usual care).

### Data collection

**Fig. 1:**
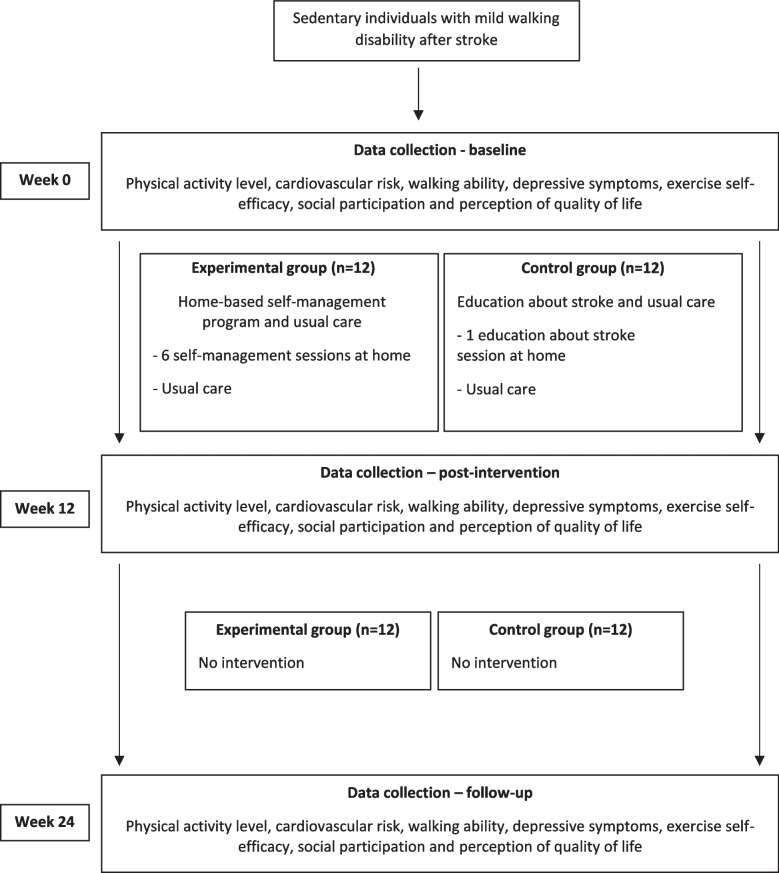
Design of the trial

Measurements will be collected in the hospital in Belo Horizonte City, Brazil, by trained researchers blinded to group allocation. Outcomes will be measured at baseline (Week 0), post-intervention (Week 12), and follow-up (Week 24) (see Fig. 1).

The measurement sessions will be restricted to 45 min. In the feasibility study, the duration of this section was 1.5 h, and participants expressed that it was too long (which may have influenced some participants’ decision to withdraw). To address this issue, we changed one instrument that measures social participation and reduced this section. We followed the SPIRIT checklist [13] to describe our protocol (see attached). The attached SPIRIT table (see Table 1) explains the schedule of this study and when enrolment, intervention/control group sessions and assessments will occur.

**Table 1:** Schedule of enrolment, intervention, and assessment

	**Enrolment**	**Allocation**	**Weeks**								**Close-out**
**TIMEPOINT**	**-t2 (1 week before data collection in week 0)**	**-t1 (1 day before data collection in week 0)**	**0**	**1**	**2**	**3**	**5**	**7**	**11**	**12**	**24**
Eligibility screen	X										
Information consent	X										
Allocation		X									
**INTERVENTION**											
Home-based self-management				X	X	X	X	X	X		
Education about stroke, orientation and usual care				X	X	X	X	X	X		
**ASSESSMENTS**											
Physical activity level			X							X	X
Cardiovascular risk			X							X	X
Walking ability			X							X	X
Depressive symptoms			X							X	X
Exercise self-efficacy			X							X	X
Social participation			X							X	X
Perception of quality of life			X							X	X

### Randomisation

A computer-generated random allocation sequence will be used. Randomisation will be used to ensure balance (half in the experimental, half in the control). Index cards sequentially numbered according to the random assignment will be printed, folded, and placed in sealed opaque envelopes. A therapist, blinded to baseline measurements, will open the envelope, assign the participant to the corresponding study group, and book the first treatment session. Then, the therapist will deliver the intervention to the experimental group and the education session to the control group. Due to the characteristics of the intervention, it will not be possible to blind the therapist and participants to group allocation.

### Intervention – experimental group

A home-based self-management program based on behaviour change techniques will be implemented through the Social-Cognitive Theory [14] and Control Theory approaches [15]. According to these theories, a behaviour to achieve a goal is influenced by self-efficacy [14] and feedback [15]. In the present study, we will use the Behaviour Change Technique Taxonomy (v1) proposed by Michie et al. 2013 to standardise the way to report *behaviour change techniques* [16]. The intervention protocol was adapted from Preston et al. 2017 and will include six sessions of home-based self-management, with an average duration of sixty minutes [17]. The content, materials, and theoretical framework of each session are described in Table 2.

**Table 2:** Intervention components, materials, theoretical framework and behaviour change techniques

Session	Components	Materials	Behaviour change technique (Theoretical framework)
**Session 1**	Education about stroke (what is, symptoms, risk factors, how to prevent another event)	Booklet “*Had a stroke, what now?*”	
**Session 2**	Feedback about initial measurement outcomes	Data from Actigraph (steps taken per day)	Feedback on behaviour (CT)
	Education about the consequences of physical inactivity	Booklet “*Consequences of physical inactivity*”	Health consequences (SCogT)
	Choose a target exercise	Exercise Preference Questionnaire	Action planning (SCogT)
	Generate a list of goals	Goal Attainment Scaling	Graded tasks (SCogT)
	Delivery self-monitoring devices	Smartband and paper-based exercise diary	Self-monitoring of behavior (CT)
**Session 3**	Review goals	Goal Attainment Scaling	Review behavior goal(s) (CT)
	Review strategies to self-monitoring	Smartband and paper-based exercise diary	Self-monitoring of behavior (CT)
	Encouraging	Verbal therapist encouragement	Non-specific encouragement (SCogT)
	Identify barriers and potential solutions	Exercise Benefits/Barriers Scale and paper-based barriers and solutions list	Problem-solving/coping planning (SCogT)
	Implementation of the physical exercise session with the participant	Paper-exercise guide	Instruction on how to perform a behaviour (SCogT)
	Development of a weekly schedule of physical exercise	Weekly activities calendar	Goal setting (behavior) (CT)
**Session 4**	Review goals	Goal Attainment Scaling	Review behavior goal(s) (CT)
	Review strategies to self-monitoring	Smartband and paper-based exercise diary	Self-monitoring of behavior (CT)
	Review weekly schedule physical exercise;	Weekly activities calendar	Goal setting (behavior) (CT)
	Encouraging	Verbal therapist encouragement	Non-specific encouragement (SCogT)
	Review barriers and potential solutions	Paper-based barriers and solutions list	Problem-solving/coping planning (SCogT)
	Vicarious experience	Paper-based stroke survivors report about the self-management program (data from feasibility study)	Vicarious reinforcement (CT)
**Sessions 5 and 6**	Review goals	Goal Attainment Scaling	Review behavior goal(s) (CT)
	Review strategies to self-monitoring	Smartband and paper-based exercise diary	Self-monitoring of behavior (CT)
	Review weekly schedule physical exercise	Weekly activities calendar	Goal setting (behavior) (CT)
	Encouraging	Verbal therapist encouragement	Non-specific encouragement (SCogT)
	Review barriers and potential solutions	Paper-based barriers and solutions list	Problem-solving/coping planning (SCogT)

Legend: *CT* Control Theory, *ScogT* Social-Cognitive Theory

Session 1 will include education about stroke (what it is, symptoms, risk factors, how to prevent another event, and orientation regarding the importance of practising physical activity after a stroke).

In Session 2, the therapist will provide feedback about sedentary behaviour and the consequences of physical inactivity. Then, the participants will choose a target exercise through the Exercise Preference Questionnaire [18] and generate a list of goals using the Goal Attainment Scale (GAS) [19]. They will be asked to set short, medium and long-term goals using the GAS. In the first moment, participants will be encouraged to set short-term goals that are important to them and with low difficulty levels. As soon as short-term goals are achieved, they will be encouraged to attain medium-term goals (medium difficulty levels). Finally, long-term goals (high difficulty levels) will be added when the medium-term goals are achieved. At the end of the session, self-monitoring devices will be provided to participants. The focus is to show participants that they can perform a behaviour and to help them achieve it by increasing self-efficacy [14] and providing them with support and feedback [15].

In Session 3, besides reviewing goals and self-monitoring strategies, the therapist will also verbally encourage all participants to motivate them to achieve a target behaviour. For those participants who completed the target behaviours, the therapist will say, “*You are doing well; let’s move on!”*. For those who have not achieved the behaviour, the therapist will say, “*Don’t give up; what can be changed for you to achieve this behaviour?*”. Then, barriers to exercise will be assessed by self-reporting and completing the Exercise Benefits/Barriers Scale [20]. The therapist, the family, and the caregivers will help each participant find solutions to the identified barriers. At the end of the session, the therapist will implement the first physical exercise session with the participant. In addition, a weekly schedule of physical exercise will be developed.

In Session 4, goals, self-monitoring strategies, and weekly physical exercise schedule will be reviewed. Barriers and potential solutions will be discussed, and verbal encouragement will be given as in the previous session. In addition, the therapist will provide a vicarious experience and show participants a paper-based report from the stroke survivors about the self-management program carried out in the previous feasibility study. This report will provide information on the barriers and benefits of this self-management program.

Sessions 5–6 will give the exact content of Session 4 except for vicarious experience.

The home-based self-management program will be delivered individually, in person, at the participant’s home, by a trained physical therapist (with knowledge of *behaviour change techniques*) over 11 weeks. The six sessions will be scheduled under the availability of the therapist and the participant’s schedule. The first three sessions will have a one-week interval between them. The following two sessions will occur at two-week intervals. Finally, the last visit will appear after a four-week break. Although the program has a standardised structure, some components of the intervention, such as choosing a target exercise, a list of goals in the GAS format, a paper guide to exercise, and a weekly physical exercise schedule, will be developed according to each participant. After exercise, participants will be asked to report any adverse events (falls, pain, etc.). To improve the recording of exercise over the feasibility study, self-monitoring devices (smartband and paper-based exercise diary) will be delivered to participants in Session 2 and will stay with them until Session 6.

Adherence to the home-based self-management exercise program will be measured by Section B of the Exercise Adherence Rating Scale [21, 22] collected at Week 11. The scores in this section range from 0 to 24; the higher the score, the higher the adherence. The answers to this scale will be based on participant self-report, reading both the smartband records and completing a paper-based exercise diary. To check adherence after the intervention, the therapist will also apply the Exercise Adherence Rating Scale at Week 24.

### Control group

The control group will receive one session of education about stroke (what it is, symptoms, risk factors, how to prevent another event, and orientation regarding the importance of practising physical activity after a stroke). This session will occur in the participant’s home, in person, on week 1, by the same therapist who will provide the intervention in the experimental group. In the subsequent weeks, they will receive usual care, which may include medical follow-up and guidance from other healthcare professionals (who are not involved with the research team) regarding the significance of behaviour change after stroke. It is a pragmatic study, so participants of both experimental and control groups will be allowed to perform daily living and healthcare activities (e.g., physiotherapy, occupational therapy, activities in a health centre, etc.).

### Primary outcome

A triaxial accelerometer will measure physical activity (Actigraph wGT3X-BT, Pensacola, FL, USA) [23]. Participants will be asked to wear the device on their waists and non-paretic sides [1]. They will be advised to wear the device during waking hours, except during water activities. The mean of steps taken per day over four days (two weekdays and two weekend days) will be recorded and reported in baseline (week 0), post-intervention (week 12) and follow-up (week 24) in both experimental and control groups. The mean of daily steps will be analysed to compare groups after intervention. As a way to describe the sample, the percentage of time spent in sedentary activities and moderate and vigorous physical activity will be reported at baseline, post-intervention, and follow-up in both groups. These data will not be used to compare groups after intervention. The Actigraph shows adequate psychometric properties in individuals after a stroke [23].

### Secondary outcomes

In the present study, we will change one questionnaire to reduce the time spent in this section. The Assessment of Life Habits Questionnaire (LIFE-H 3.1) [24], which measures social participation, will be replaced by the Stroke Impact Scale [25].

- Cardiovascular risk will be measured according to the American College of Sports Medicine as body mass index (kg/m2), waist circumference (cm), and blood pressure (mmHg) [26].
- Depressive symptoms will be measured by the short version of the Geriatric Depression Scale [27]. The results will be reported in scores from 0 to 15, where scores ≥ 6 suggest the presence of depression [27]. This scale shows adequate psychometric properties in stroke [28].
- Walking ability will be measured by the distance covered in the 6-Minute Walk Test and by the habitual and maximum walking speed through the 10-Meter Walk Test. They will be implemented following previous recommendations [29]. These tests show adequate psychometric properties in stroke [30].
- Exercise Self-Efficacy will be measured through the Self-Efficacy for Exercise scale. The results will be reported in scores from 0 to 10, where 10 is the maximum self-efficacy [31]. This scale shows adequate psychometric properties in stroke [31].
- Social participation will be measured by the participation domain of the Stroke Impact Scale, the Brazilian version [25]. The results will be reported in percentage from 0 to 100%, where a higher percentage demonstrate better social participation levels. This scale shows adequate psychometric properties in stroke [25].
- Quality of life will be measured by the Brazilian version of EuroQual-5D [32]. The results will be reported in scores from 0–100, where higher scores demonstrate a better quality of life [32]. This scale shows adequate psychometric properties in stroke [32].

### Data monitoring body

The overall responsibility for the trial is under the supervision of Aline Scianni. The responsibility for stopping recruitment in case of several serious adverse events is under the control of Christina Faria. She is a researcher who is not involved in the present study. She will receive reports on adverse events for each inclusion of 5 participants.

### Sample size estimate

The present study is a Phase II pilot clinical trial and a specific intervention in a particular sample [sedentary individuals (> 5,000 steps/day) and with mild walking disability (gait speed ≥ 0.8 m/s] will be developed. So, data from the mean and standard deviation required to produce a sample size are unavailable in the literature. So, in this case, a formal sample size calculation will not be undertaken. To estimate the number of participants required in the present study, we will follow the recommendations of Julious et al., who support that 12 subjects per group are enough for pilot clinical trials [33, 34].

### Statistical analyses

All analyses will be conducted intention-to-treat by a statistician blinded to group allocation. Descriptive analyses will be used as mean and standard deviation for parametric data and median and interquartile interval for non-parametric data. The analysis will include examining data distribution and variance equality to confirm the appropriate application of parametric analysis. Information from the latest available evaluation will be used for missed sessions. Two-way analyses of variance with repeated measures at the three-time points (baseline, post-intervention, and follow-up) will be implemented for all parametric outcomes to determine the statistical significance of the between-group differences and reported as mean differences between groups (95% CI) [35]. For non-parametric outcomes, the Kruskal–Wallis test will be used to determine the statistical significance of the between-group differences and reported as mean differences between groups (95% CI) [35]. Baseline characteristics judged clinically different between groups may be included as covariates and will be analysed using analysis of covariance (ANCOVA) and presented as mean differences between groups (95% CI) [35]. All analyses will be performed using the SPSS software (23.0 version).

## Discussion

Although self-management programs have been used to promote physical activity after stroke, the effectiveness of this strategy is still unclear [5, 6]. In this way, further studies in the area are required [4, 7, 8]. Recently, we demonstrated, in a Phase I study, the feasibility of implementing a home-based self-management exercise program to promote physical activity among sedentary stroke survivors with mild walking disability in Brazil [1]. In conclusion, our feasibility study showed that a Phase II study is required. However, some barriers to implementing the next phase were found in this previous study (i.e. lack of medical clearance for participants to practice physical activity, too much time spent in the data collection section, difficulty in acquiring information about exercise adherence, etc.). Therefore, in the present Phase II study, we will address these previously encountered barriers and, in addition, test this program against a control group. If beneficial, the results of this study will provide data to plan a fully powered future Phase III clinical trial aimed at verifying the program’s efficacy in promoting physical activity after stroke.

This study has some limitations. First, since a sample size calculation will not be made, the interpretation of results must be taken with caution. Second, this is a Phase II trial, which aims to determine whether an intervention has potential benefits to justify further investigation in a Phase III trial [36]. Thus, the results from the present study cannot be used to change clinical practice [36]. For this, more investigation is necessary in the next trial phase. The strengths of the present study are that if beneficial, we will provide data to plan a fully powered future Phase III trial. Besides, we will raise barriers to implementing the intervention in the next Phase, which can increase the probability of success in a future study. If not beneficial, we will avoid spending time and expenses on a large study.

### Supplementary Information

**Additional file 1.**

## Data Availability

The data and materials associated with the manuscript will be available upon reasonable request.

## Abbreviations

CI: Confidence interval
GAS: Goal Attainment Scale
kg/m2: Kilogram per square meter
mmHg: Millimetre of mercury
